# The complete chloroplast genome and phylogenetic position of *Thamnocalamus unispiculatus* (Poaceae: Bambusoideae: Arundinarieae)

**DOI:** 10.1080/23802359.2022.2088308

**Published:** 2022-06-28

**Authors:** Weihua Wang, Shiyu Lv, Li Liu, Yuanyan Meng, Xiaolong Zhang, Xiaying Ye

**Affiliations:** a Agronomy and Life Science Department, Zhaotong University, Zhaotong, China; b Research Center for Plateau Characteristic Agriculture in Northeast Yunnan, Zhaotong University, Zhaotong, China; c Germplasm Bank of Wild Species, Kunming Institute of Botany, Chinese Academy of Sciences, Kunming, China

**Keywords:** Arundinarieae, Bambusoideae, chloroplast genome, phylogeny, *Thamnocalamus unispiculatus*

## Abstract

*Thamnocalamus unispiculatus* T.P.Yi & J.Y.Shi 2007 is an important bamboo species with significant ecological and economic value. This study presents the complete chloroplast genome sequence of *T. unispiculatus*. The sequence was 139,726 bp in length and exhibited a typical quadripartite structure, containing four regions: large single copy regions (LSC, 83,283 bp), small single copy regions (SSC, 12,851 bp) and a pair of inverted repeats (IRs, 21,726 bp). A total of 130 genes were annotated, including 86 protein-coding genes, 36 transfer RNA genes, and eight ribosomal RNA genes. Phylogenetic analysis indicated that *T. unispiculatus* and *T. spathiflorus* are sister species, supporting the conclusion that *Thamnocalamus* is a monophyletic group. The chloroplast genome of *T. unispiculatus* promotes the protection and exploration of biodiversity, phylogenetic relationships, and genetic research in Bambusoideae.

*Thamnocalamus* Munro is a Himalaya-centered genus comprising two to four species; only one species occurs in China (Li et al. 2006). Chloroplast DNA fragments and chloroplast genome analyses have demonstrated that this genus is of monophyletic lineage and is named as clade VII (Zhang et al. 2012; Attigala et al. 2016; Zhang et al. 2016; Ma et al. 2017). However, only *Thamnocalamus spathiflorus* (Trinius) Munro 1868 and *T. spathiflorus* var. *crassinodus* (T.P.Yi) Stapleton 1994 have been investigated in previous studies, lacking of supporting evidence from other species. *T. unispiculatus* T.P.Yi & J.Y.Shi 2007 was described in 2007 and named “niu se ma” in Tibetan typically (Yi et al. 2007). The discovery of *T. unispiculatus* provides an opportunity to verify the monophyly of *Thamnocalamus* and clarify its intrageneric relationship.

*T. unispiculatus* is an important germplasm resource with strong resistance to freezing temperatures and soil erosion, largely occurring in the alpine zone at altitudes between 2600 m and 3600 m in southwest Tibet, China. Moreover, its shoots are rich in nutrients, containing high-quality proteins, essential amino acids, bioactive compounds, and dietary fiber (Singhal et al. 2013), which is of great economic value. *T. unispiculatus* also plays an important role in maintaining ecosystem function, providing crucially food and habitat for rare endangered fauna, including the red panda. However, the chloroplast sequence of *T. unispiculatus* has not been annotated and reported yet. This study aimed to clarify the complete chloroplast genome of *T. unispiculatus*. The outcomes could promote further research on phylogenetic relationships, germplasm protection and exploration, and molecular biology of Bambusoideae.

Leaf materials and specimens of *T. unispiculatus* were collected from Jirong, Tibet, China (28°32'29″ N and 85°13'42″ E; 3504 m altitude) and the voucher specimens were deposited in the Herbarium of Kunming Institute of Botany, Chinese Academy of Sciences (KUN, http://www.kib.cas.cn/; contact person and email: Ye Xia-Ying, 34007@ztu.edu.cn; accession number: YXY241). Genomic DNA was extracted from dried leaf tissue using a modified cetyltrimethylammonium bromide (CTAB) method (Doyle 1987), in which 4% CTAB was used and 0.1% DL-dithiothreitol was added. High-quality DNA was fragmented to construct libraries (500 bp) for genome skimming sequence using an Illumina HiSeq 2000 (Illumina, San Diego, United States) at BGI, in Shenzhen, China. Approximately 2 Gb of data was obtained for each sample. The plastome was assembled using the GetOrganelle pipeline (-F embplant_pt; -k 35, 45, 55, 65, 85, 105) (Jin et al. 2020). The gaps in the scaffold were filled using GapCloser with default parameters, a package belonging to the novel short-read assembly method SOAPdenovo2 (https://sourceforge.net/projects/soapdenovo2/files/GapCloser). All paired-end clean reads were mapped to the assembled chloroplast genome and the coverage exceeded 230×. Finally, the assembled chloroplast genome sequence was annotated using the Plastid Genome Annotator (PGA) pipeline with default parameters followed by manual correction (Qu et al. 2019). *T. spathiflorus* (NC_024724.1) was selected as the reference.

The chloroplast genome of *T. unispiculatus* (GenBank accession: NC_061040.1) was 139,726 bp in length with a guanine-cytosine (GC) content of 38.82%. The genome contained a large single copy region (LSC, 83,283 bp), a small single copy region (SSC, 12,851 bp), and a pair of inverted repeats (IRs, 21,726 bp). The plastome comprised 130 genes in total; this included 86 protein-coding genes, 36 transfer RNA (tRNA) genes, and eight ribosomal RNA (rRNA) genes. Eight protein-coding genes (*rps7, rps12, rps15, rps19, rpl2, rpl23, ndh*B, and *ycf68*), eight tRNA genes (*trn*H-GUG, *trn*I-CAU, *trn*L-CAA, *trn*V-GAC, *trn*I-GAU, *trn*A-UGC, *trn*R-ACG, and *trn*N-GUU), and four rRNA genes (*rrn16*, *rrn23*, *rrn4.5*, and *rrn5*) were duplicated in the IRs.

To determine the phylogenetic position of *T. unispiculatus*, a maximum-likelihood phylogenetic tree was constructed based on 25 complete chloroplast genomes of Arundinarieae using the MAFFT v. 7.471 (Katoh and Standley 2013) and RAxML v. 8.2.12 (Stamatakis 2014) programs. The CIPRES Science Gateway (Miller et al. 2010) was utilized to conduct the analyses, default parameters were set and *Hsuehochloa calcarea* (C.D.Chu & C.S.Chao) D.Z.Li & Y.X.Zhang was selected as the outgroup (Zhang et al. 2018). The phylogenetic tree showed that *T. unispiculatus* and *T. spathiflorus* are sister species with strong support (100%), and *Thamnocalamus* constituted a monophyletic group (Figure 1); this was consistent with previous research results (Attigala et al. 2016; Ma et al. 2017; Guo et al. 2021).

**Figure 1.**
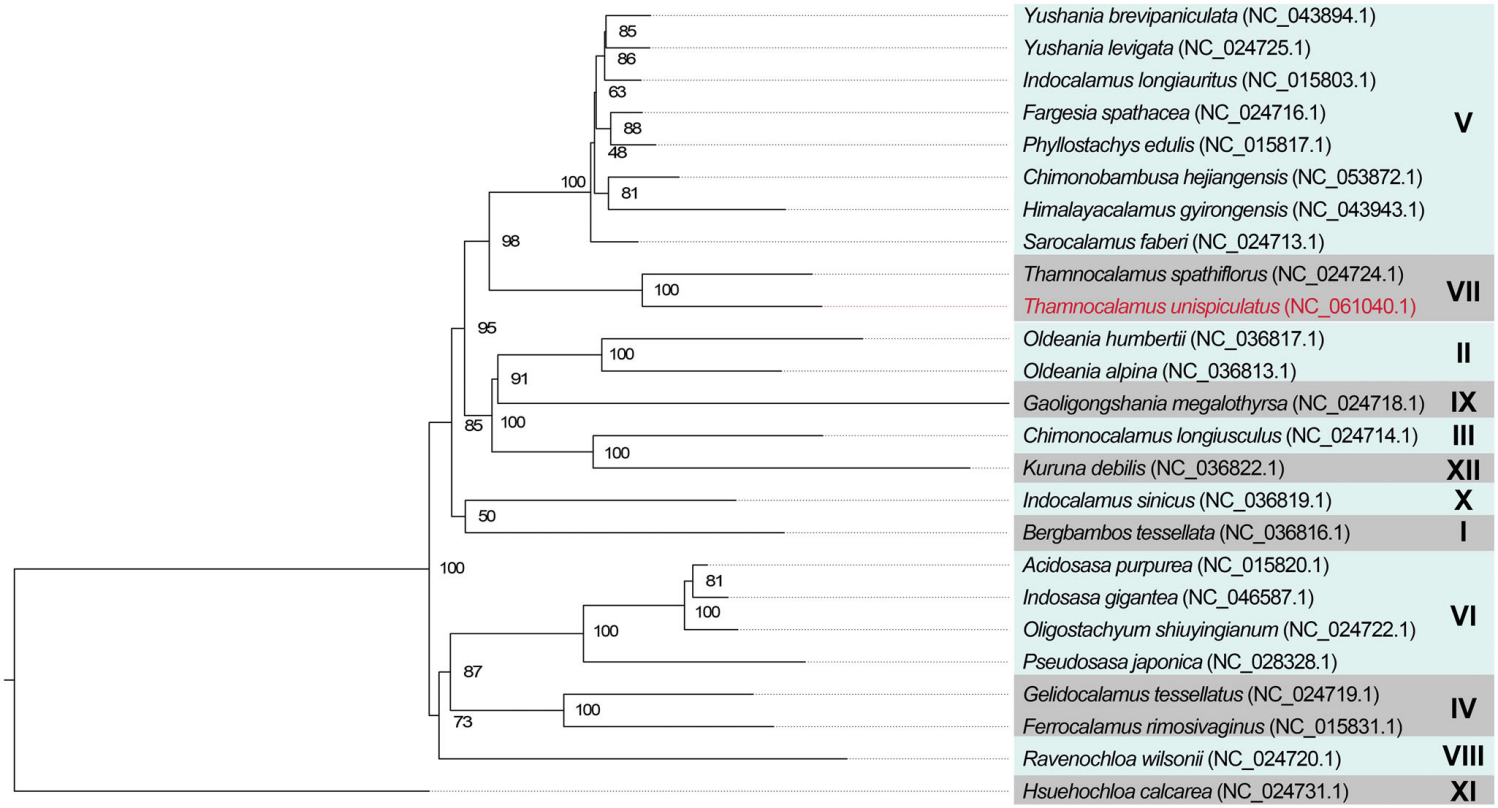
Maximum-likelihood phylogenetic tree showing relationship between *Thamnocalamus unispiculatus* (red color) and other 24 species in Arundinarieae, *Hsuehochloa calcarea* (C.D.Chu & C.S.Chao) D.Z.Li & Y.X.Zhang (NC_024731.1) was selected as outgroup. Numbers in each node indicated the bootstrap support values. Roman numerals represent the clade name established in previous studies.

## Ethical statement

The collection of *Thamnocalamus unispiculatus* T.P.Yi & J.Y.Shi was carried out in accordance with guidelines provided by Kunming Institute of Botany, Chinese Academy of Science and national regulations.

## Data Availability

The genome sequence data that support the findings of this study are openly available in GenBank of NCBI at (https://www.ncbi.nlm.nih.gov/) under the accession No. NC_061040.1. The associated Bio-Project, Bio-Sample and SRA numbers are PRJNA773614, SAMN22502075 and SRR16530667 respectively.
